# Evaluating a tylosin dosage regimen for treatment of *Staphylococcus delphini* infection in mink (*Neovison vison*): a pharmacokinetic-pharmacodynamic approach

**DOI:** 10.1186/s13567-021-00906-0

**Published:** 2021-02-27

**Authors:** Amir Atabak Ronaghinia, Julie Melsted Birch, Henrik Lauritz Frandsen, Pierre-Louis Toutain, Peter Damborg, Tina Struve

**Affiliations:** 1 Department of Veterinary and Animal Sciences, University of Copenhagen, Stigbøjlen 4, 1870 Frederiksberg C, Denmark; 2 Department of Veterinary and Animal Sciences, University of Copenhagen, Ridebanevej 3, 1870 Frederiksberg C, Denmark; 3 National Food Institute, Technical University of Denmark, Kemitorvet, Building 204, 2800 Kongens Lyngby, Denmark; 4 Kopenhagen Diagnostics, Department of Health and Diagnostics, Kopenhagen Fur a.m.b.a., Langagervej 60, 2600 Glostrup, Denmark; 5 Royal Veterinary College, University of London, Hawkshead Campus, Hatfield, AL9 7TA UK; 6 INTHERES, Université de Toulouse, INRA, ENVT, 23 Chemin des Capelles, BP 87614, 31076, Toulouse Cedex 3, France

## Abstract

*Staphylococcus delphini* is one of the most common pathogens isolated from mink infections, especially dermatitis. Tylosin (TYL) is used frequently against these infections, although no evidence-based treatment regimen exists. This study aimed to explore the dosage of TYL for infections caused by *S. delphini* in mink. Two animal experiments with a total of 12 minks were conducted to study the serum pharmacokinetic (PK) characteristics of TYL in mink after 10 mg/kg IV and oral dosing, respectively. The concentration of TYL in serum samples collected before and eight times during 24 h after TYL administration was quantitated with liquid chromatography quadrupole time-of-flight mass spectrometry, and the TYL disposition was analyzed using non-linear mixed effect analysis. The pharmacodynamics (PD) of TYL against *S. delphini* were studied using semi-mechanistic modeling of in vitro time-kill experiments. PKPD modeling and simulation were done to establish the PKPD index and dosage regimen. The disposition of TYL was described by a two-compartmental model. The area under the free concentration–time curve of TYL over the minimum inhibitory concentration of *S. delphini* (*f*AUC/MIC) was determined as PKPD index with breakpoints of 48.9 and 98.7 h for bacteriostatic and bactericidal effect, respectively. The calculated daily oral dose of TYL was 2378 mg/kg, which is 238-fold higher than the currently used TYL oral dosage regimen in mink (10 mg/kg). Accordingly, sufficient TYL concentrations are impossible to achieve in mink plasma, and use of this drug for extra-intestinal infections in this animal species must be discouraged.

## Introduction

*Staphylococcus delphini* is one of the most common bacterial pathogens in mink. As part of the mink mucosal and integumentary microbiota, this is an opportunistic pathogen that may infect different organs, especially skin [1]. For example, pododermatitis (footpad infection) is a common manifestation of this agent causing discomfort, deterioration of fur quality and lower breeding rates in mink farms [2, 3]. Tylosin (TYL) is a veterinary macrolide often used empirically for skin infections in mink despite the absence of an evidence-based treatment regimen. TYL inhibits bacterial protein synthesis and is active against Gram-positive and to a lesser extent Gram-negative bacteria [4]. To the authors’ knowledge, there are no pharmacokinetic (PK) or pharmacodynamic (PD) data available for this drug in mink. Therefore, following the European cascade principle [5], the currently used daily oral dosage (typically 10 mg/kg) has been adapted from that used in other domestic species, i.e. pig and cattle. The objectives of this study were to (i) investigate PK parameters of TYL in mink after intravenous (IV) and oral (PO) administration, (ii) characterize PD parameters of TYL against *S. delphini*, (iii) predict a target value for the TYL PKPD index in mink [6], and (iv) use the obtained data to establish a TYL dosage regimen in mink.

## Materials and methods

### Animal experiments

PK characteristics of TYL after IV and oral administration were investigated in two animal experiments with a total of 12 healthy brown male minks (*Neovison vison*) purchased from a commercial farm. In the first animal experiment, drug disposition after IV administration was studied in six minks. Each animal was subjected to cephalic IV injection of 10 mg/kg TYL (tylosin 20%, Tylan^®^ Vet., Elanco Animal Health, Herlev, Denmark), and paired blood samples were collected under anesthesia (see “Blood sampling” section) just before and eight times after antibiotic administration (15, 30, 60 and 120 min, and 6, 10, 15 and 24 h) from a cephalic vein and from nails. In the second animal experiment, TYL disposition following oral administration of 10 mg/kg TYL (tylosin phosphate 10%, Tylan^®^ Vet., Elanco Animal Health, Herlev, Denmark) mixed into 135–150 g feed (0.2 g/kg) was explored in six minks. In order to ensure rapid consumption of the feed mix, minks had been fasted for 18 h prior to medication. Blood samples were collected before and eight times after antibiotic administration (30, 60, 120 min and 6, 10, 15, 24 and 48 h) by nail clipping. Prior to both experiments, minks had been acclimatized for 7–10 days with access to ad libitum water and 250–300 g commercial feed per day in standard commercial cages (1^st^ experiment) and in metabolic cages (2^nd^ experiment), the latter of which differed by having a feed bowl but no nest attached. Following the Danish order BEK nr 856 of 27.06.2013 § 1, each cage was equipped with a water bottle, a plastic shelf and a plastic tube as enrichment. After obtaining all blood samples, minks from the IV experiment were euthanized by intracardiac injection of pentobarbital sodium (Euthasol^®^, Virbac Danmark, Kolding, Denmark) while under inhalation anesthesia. Minks from the PO experiment were euthanized by carbon dioxide inhalation following intramuscular injection of ketamine (Ketaminol^®^, MSD Animal Health, Copenhagen, Denmark) and xylazin (Rompun^®^, Bayer, Copenhagen, Denmark).

### Anesthesia and analgesia

In the first animal experiment, IV administration of TYL and the initial five blood samplings were conducted during one continuous up to 3 h period of inhalation anesthesia using sevoflurane (Sevoflo^®^, Orion Pharma, Copenhagen, Denmark) [7]. Each of the subsequent blood samples were collected during a short period of sevoflurane anesthesia (15–35 min). Anesthesia was induced in an induction chamber with 8% sevoflurane for 3–5 min after 8–10 min of pre-oxygenation (∼90% O_2_). For maintenance, sevoflurane (2.5–5%, depending on monitored vital signs) was administered through a laryngeal mask (V-gel^®^, Docsinnovent, London, UK). Heart rate, respiration rate, body temperature, non-invasive blood pressure, O_2_ saturation, hemoglobin in arterial blood, and end tidal CO_2_ concentration were recorded every 15 min during anesthesia using pulse oximetry and a multifunction anesthesia monitor (Datex Ohmeda, WI, USA). Anesthesia was not applied in the second experiment, as all blood samples were collected from the nails of entrapped awake mink. As an analgesic, 0.2 mg/kg meloxicam (Metacam, Boehringer Ingelheim International, Germany) was administered subcutaneously immediately before the first blood sampling in both experiments. After nail clipping, ferric chloride hexahydrate (50%) (Sigma-Aldrich, MO, USA) was applied topically for hemostasis.

### Blood sampling

Blood samples (500–800 µL) were collected in capillary tubes (KABE LABORTECHNIK, Elsenroth, Germany) containing a coagulation inducer and a separating gel. After 10 min at room temperature, to induce clot formation, tubes were centrifuged at 1000 *g* for 10 min followed by storage of the supernatant (> 150 µL serum) at −80 °C until further analysis.

### Serum protein binding rate of TYL

The binding of TYL to plasma proteins was quantified by an in vitro ultrafiltration method [8]. Prior to the study, fifty mL pooled serum from 10 different minks in a commercial farm had been collected during pelting by heart puncture immediately after CO_2_-mediated euthanasia. TYL (Merck, Darmstadt, Germany) was added to thawed serum samples at concentrations of 5, 50 and 500 µg/mL. As a negative control, TYL was added to PBS. The sera were incubated at 38 °C for 60 min to mimic the body temperature of mink. Samples were then filtered using 30 kDa filter tubes (Amicon^®^ Ultra-2, 30 kDa; Merck Millipore) with 4000 *g* centrifugation for 20 min at room temperature. Filtered aliquots were stored at −80 °C until further analysis. All experiments were conducted in triplicate. To remove glycerin residues from tubes before the experiment, they were washed once with 0.5 mL of 0.1 M NaOH, rinsed twice with 1.0 mL PBS, and centrifuged at 7000 *g* for 20 min after each rinse/wash. After quantification of TYL in each sample, a linear regression curve was used to fit the mean value of each concentration.

### Quantification of TYL in samples

A liquid chromatography (LC) method with quadrupole time of flight mass spectrometry detection (QTOF-MS) was used for measuring the concentration of TYL in serum and PBS samples from the animal experiments and the serum protein binding assay. The detailed procedure is presented in Additional file 1. In brief, matrix-matched calibration samples were prepared in blank serum at seven different concentrations in the range of 4–500 ng/mL. The standard calibration curves were analyzed at the beginning, after each 20 samples and at the end of the sequence. Extracted ion chromatograms of m/z 916.5264 (± 0.005) were constructed and integrated. No interfering peaks were observed at the retention times of the antimicrobials. Serum concentrations were calculated based on linear calibration curves constructed using 1/x weighing. As all samples were analyzed in one sequence, the inter-day precision is not relevant, and the intra-day precision (CV%) for samples was < 10%. The lower limit of quantification (LLOQ) was 30 ng/mL for TYL. Any data lower than the LLOQ were flagged as Below Quantification Limit (BQL) for data analysis.

### In vitro antimicrobial growth (time-kill) experiment

Time-kill experiments were done using two wild-type clinical *S. delphini* isolates (16–12727-1, 16–12403-3) of mink origin after measuring their TYL MIC by broth microdilution according to the Clinical Laboratory Standards Institute [9]. Three to five single colonies from fresh Mueller Hinton agar (MHA) plates were incubated for 2 h in 5 mL Mueller Hinton broth (MHB) at 37 °C with shaking (150 rpm) to reach exponential growth phase. After incubation, bacterial concentration was adjusted to ~10^8^ CFU/mL, and 10 µL of this aliquot was inoculated in 2 mL tubes containing 1 mL of MHB supplemented with TYL, followed by incubation at 37 °C with gentle shaking (150 rpm). The TYL concentrations used in time-kill assays corresponded to 8, 4, 2, 1, 0.5 and 0.25-fold the MIC values of the two *S. delphini* isolates used for the experiment. Twenty µL aliquots removed at times 0, 1, 2, 4, 8 and 24 h post-inoculation were tenfold serially diluted in saline followed by plating 4 × 10 µL of each dilution on MHA plates in duplicate for determination of viable counts upon overnight incubation at 37 °C. The LLOQ for the bacterial count was 200 CFU/mL, and data lower than this limit were flagged as BQL for data analysis. The average of viable colony counts was used for analysis.

### PD modeling of in vitro antimicrobial growth curves

**Figure 1:**
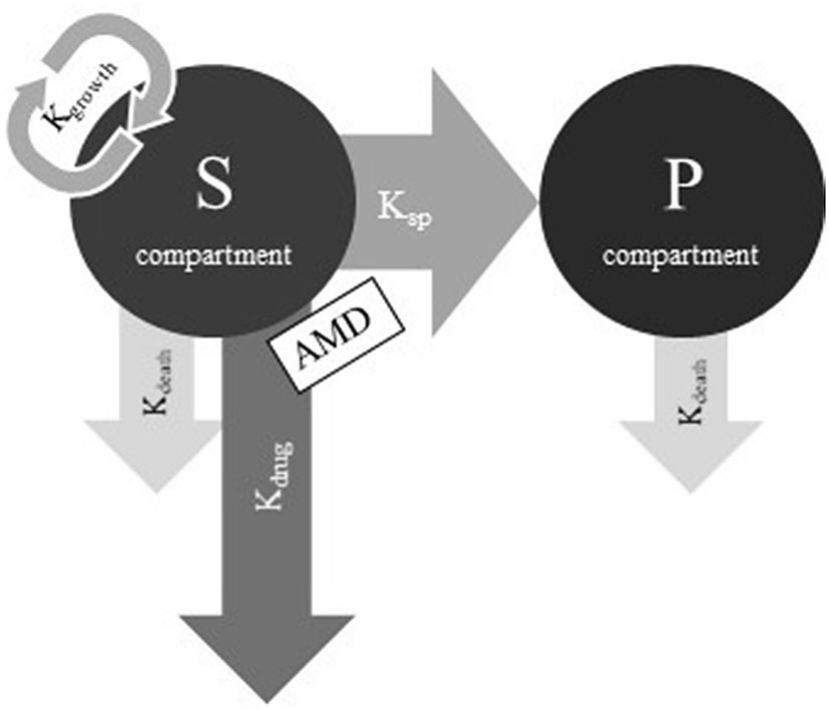
**Semi-mechanistic model used for PD modelling of time kill data.** The S compartment contains the susceptible bacterial population, the P compartment contains the non-susceptible bacterial population, K_growth_ and K_death_ are the bacterial growth and natural death rate constants, respectively, K_sp_ is the transformation rate constant between S and P compartments, K_drug_ is the drug kill rate constant caused by exposure to an antimicrobial drug (AMD) [15].

A previously described semi-mechanistic model (Figure 1) was used for PD modeling of time-kill data [10]. In summary, the model consists of an S and a P compartment containing a susceptible and a non-susceptible bacterial population, respectively. The bacterial growth in S (1) is assumed to be regulated by the natural growth rate, the natural death rate and the kill rate of an antimicrobial drug.

1 $${\raise0.7ex\hbox{${dS}$} \!\mathord{\left/ {\vphantom {{dS} {dt}}}\right.\kern-\nulldelimiterspace} \!\lower0.7ex\hbox{${dt}$}} = K_{growth} \times S - (K_{death} + K_{drug} ) \times S - K_{SP} \times S$$

where S (CFU/mL) is bacterial concentration in the S compartment, t (h) is time, and K_growth_, K_death_ and K_drug_ (1/h) are rate constants of bacterial growth, bacterial natural death, and bacterial kill by TYL, respectively. K_SP_ (1/h) is a rate constant describing the rate of transfer from the S to the P compartment. K_SP_ is a linear function of total bacterial concentration (S + P) in the system (2).

2 $$K_{SP} = \frac{{\left( {k_{growth} - K_{death} } \right)}}{{B_{max} }} \times \left( {S + P} \right)$$

where B_max_ (CFU/mL) is the highest achievable bacterial concentration in the system. It is assumed that the bacterial population in the P compartment has no growth, is non-susceptible to TYL, and has the same natural death rate as in the S compartment (3).

3 $${\raise0.7ex\hbox{${dP}$} \!\mathord{\left/ {\vphantom {{dP} {dt}}}\right.\kern-\nulldelimiterspace} \!\lower0.7ex\hbox{${dt}$}} = K_{SP} \times S - K_{death} \times P$$

The effect of TYL is assumed to follow a non-linear function that depends on the concentration in the system and is described by an E_max_ sigmoid model (4).

4 $$K_{DRUG\left( t \right)} = \frac{{Emax \times C\left( t \right)^{\gamma } }}{{EC_{50}^{\gamma } + C\left( t \right)^{\gamma } }}$$

where E_max_ (1/h) is maximum bacterial kill by TYL representing drug efficacy, EC_50_ (mg/L) is the concentration of TYL that produces half of the maximum effect measuring drug potency, gamma (γ-scalar) is a sigmoidicity coefficient expressing the slope of antimicrobial effect curves and presenting drug sensitivity, and C(t) is the concentration of TYL at time (t).

In addition, the MIC (5) and the minimum bactericidal concentration (MBC) (6) of TYL for *S. delphini* were estimated directly from the estimated parameters of the model as secondary parameters [11].

5 $$MIC = EC_{50} \times \left( {\frac{{K_{Growthnet} - 0.26}}{{Emax - \left( {K_{Growthnet} - 0.26} \right)}}} \right)^{\frac{1}{Gamma}}$$

6 $$MBC = EC_{50} \times \left( {\frac{{K_{Growthnet} + 0.288}}{{Emax - \left( {K_{Growthnet} + 0.288} \right)}}} \right)^{\frac{1}{Gamma}}$$

where K_Growthnet_ is the net bacterial growth (i.e. K_Growthnet_ = K_Growth_ – K_death_).

The drug was assumed to possibly undergo an in vitro degradation over the duration of the test, and a parameter reflecting this possible decay (K_el_) was added to empirically improve the model fit (no experimental data). Since our analysis revealed no evidence of such decay over 24 h, K_el_ was fixed to 0. The estimation of parameters were obtained using a non-linear mixed effect analysis (NLME-Phoenix^©^ 8.3 software package, Certara, NJ, USA), which was based on minimizing an objective function value (OFV) by the population Laplacian engine of Phoenix^®^. Since large variability was expected for B_max_, the random component was added to this parameter for fitting purposes. BQL data were treated as censored with the M3 method [12]. The parameter mean and precision estimates were obtained by using the bootstrap tool (*n* = 30 replicates) in Phoenix 8.3.

### PK modeling of in vivo concentration–time data

A non-linear mixed effect (NLME) analysis of TYL concentration–time data was done using the Phoenix^©^ 8.3 software package. The objective of this analysis was to estimate the TYL PK parameters and their between subject variability (BSV) in the population after IV and oral dosing. The data obtained following IV and oral administration were analyzed simultaneously and fitted to the one and two-compartmental structural model. Determination of the best model (one- or two-compartmental) was based on visual inspection of plots and Akaike’s information criterion (AIC). The variance of parameters across individuals (BSV) was computed based on an exponential model. The variance of parameters has a log-normal distribution and was converted to coefficient of variation (CV) in original scale (7).

7 $${\text{CV}}\left( {\text{\% }} \right) = 100 \times \sqrt {\exp \left( {{\upomega }^{2} } \right) - 1}$$

where omega (ω^2^) is the variance of parameters across individuals.

The random component of the parameter was removed from the model, when shrinkage of random effects toward the means was high (> 0.4). The residual error for parameters was modeled using the additive and multiplicative model. As the dose was computed by using plasma clearance, the influence of the sampling site (vein vs. nail) on this PK parameter was tested by comparing the value of the Bayesian Information Criterion (BIC) of the two models with and without including the sampling site as a covariate for the clearance. The censored data (BQL) were handled using the M3 method [12]. A Laplacian engine was used in the estimation process, and the parameters mean and precision were computed using a bootstrap tool (*n* = 30).

### Prediction of the TYL PKPD index

A previously described PKPD model was used in this study [13]. In brief, the in vivo TYL disposition following oral administration was integrated into the in vitro PD model described in Section “PK modeling of in vivo concentration–time data”. The PK mono-exponential part of the in vitro model (i.e. K_el_ = 0) was replaced by the actual in vivo PK disposition model (see Section “PK modeling of in vivo concentration–time data”) to predict the bacteriological effect of TYL. To estimate the PKPD target, simulations of the time development of in silico microbiological load were applied with a bacterial initial load of 5 × 10^5^ CFU/mL at four MIC levels (0.125, 0.25, 0.5 and 1 mg/L). The wide range of 13 daily doses of TYL corresponding to twofold increases of concentration from 1 × MIC to 2048 × MIC for *S. delphini*, providing 52 paired datasets (i.e. 13 daily doses for each of 4 MIC levels) were simulated for each of two PKPD indices, namely time above the MIC (*f*T(%) > MIC) and Area Under the Concentration–time curve over the MIC (*f*AUC/MIC). It was assumed that the efficacy (E_max_) did not change with MIC and the difference in MICs was solely dependent on TYL potency (EC_50_). Hence, a scaling factor for the EC_50_, obtained from the in vitro PD model, was used to convert measured MIC (0.25 mg/L) to the simulated MIC. For the bacteriological effect, Log_10_ transformation of the cumulative area under the curve of the total bacterial concentration over 24 h (Log_10_AUC_totbact_) was used. Bacteriostatic and bactericidal effects were defined as no change and one Log_10_ reduction in the initial inoculum, respectively. The area under the free serum concentration–time curve (*f*AUC_po,0−24_), with 69% free drug (*f*), was obtained directly from the PK model (see Section “PK modeling of in vivo concentration–time data”), and percentage of time that serum concentration exceeded MIC within 24 h (*f*T(%) > MIC) was computed using the non-compartmental and statistical tools of the Phoenix^©^ software package. The 52 datasets of *f*T(%) > MIC (independent variable) versus AUC_totbact_ (dependent variable) and *f*AUC_po,0–24_/MIC (independent variable) versus AUC_totbact_ (dependent variable) for each bacterial strain were fitted individually for each MIC with an inhibitory sigmoid I_max_ model (8).

8 $$E = E_{0} - \frac{{I_{max} \times Index^{\gamma } }}{{Index_{50}^{\gamma } + Index^{\gamma } }}$$

where E_0_ is no effect of TYL (obtained from the control curves for C(t) = 0), I_max_ is extent of the maximal effect, Index_50_ is magnitude of the indices (*f*AUC_po,0−24 h_/MIC or *f*T(%) > MIC) achieving 50% of the I_max_, and γ is the sigmoidicity factor reflecting the steepness of the relationship. The maximum observed effect was calculated by subtracting I_max_ from E_0_ (i.e. E_0_-I_max_). Curve fitting was performed in WinNonlin^®^ (Certara, NJ, USA). The coefficients of determination (R^2^), the AIC, and visual inspection of plots were used to select the PKPD index best describing the antimicrobial effect.

### TYL dosage prediction

After obtaining PK and PD parameters, the first dosage regimen was calculated using the following Eq. (9):

9 $$Dosage_{per day} = \frac{{Cl_{day} \times SF \times ECOFF}}{F \times f}$$

where Cl_day_ (L/day/kg) is the TYL body clearance in 24 h, SF (scalar) is the scaling factor obtained by dividing the selected PKPD index i.e. *f*AUC_po,0−24 h_/MIC by 24 h, F is bioavailability, *f* is free fraction of drug in serum and ECOFF is the highest MIC for organisms devoid of phenotypically-detectable acquired resistance mechanisms. In this study, we used the recently determined TYL tentative ECOFF (TECOFF) of 2 mg/L for *S. delphini* [14].

### Statistical analysis

PK and PD parameters from NLME are presented as typical values of the population with corresponding precision as standard errors.

## Results

### Serum protein binding rate of TYL

The free fraction of TYL was obtained as the ratio of the slope coefficient b of the spiked serum curve to the control solution curve, multiplied by 100. According to the results, the mean free fraction of TYL in mink serum was 69%.

### Antimicrobial growth (time-kill) experiment

**Figure 2:**
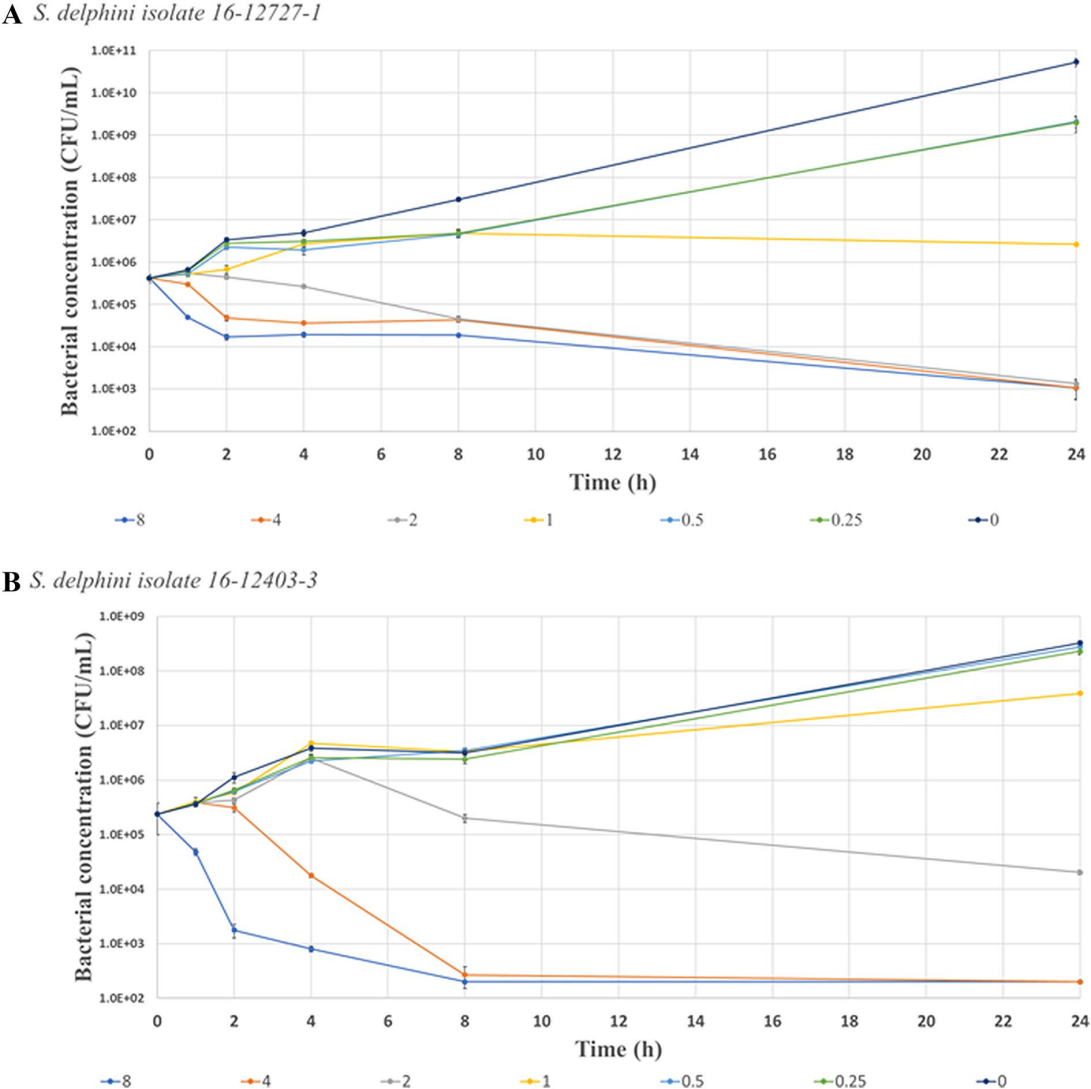
**Time kill curves for TYL vs. two isolates of S. delphini (A, B) in MHB over 24 h**. The Y-axis presents the bacterial concentration starting from LLOQ as 200 CFU/mL. The X-axis is the time post-inoculation. The error bar at each time point presents the SD for duplicates. Different colors depict the various TYL concentrations with two-fold increase at each step corresponding to 0.25, 0.5, 1, 2, 4 and 8 × MIC. The MIC for both isolates was 0.25 mg/L. The concentrations higher than 2 × MIC resulted in bacterial kill in both isolates. In plot (**A**), the concentrations of 0.5 and 4 × MIC are masked by 0.25 and 2 × MIC, respectively. In plot (**B**), the concentrations of 0.25 and 0.5 × MIC are masked by the control (0 × MIC).

The MIC of the two *S. delphini* isolates used in the time kill experiment was 0.25 mg/L. Figure 2 presents the time-kill curves of TYL for these *S. delphini* isolates grown in MHB with two-fold increasing TYL concentrations corresponding to 0.25, 0.5, 1, 2, 4 and 8 × MIC. At 0.25 and 0.5 × MIC, bacterial growth continued until reaching the stationary phase. At the two highest TYL concentrations, a three log reduction in bacterial concentration was observed at 8 h or at 24 h post-inoculation depending on the isolate tested (Figure 2).

### PD modeling of time-kill experiments

**Figure 3:**
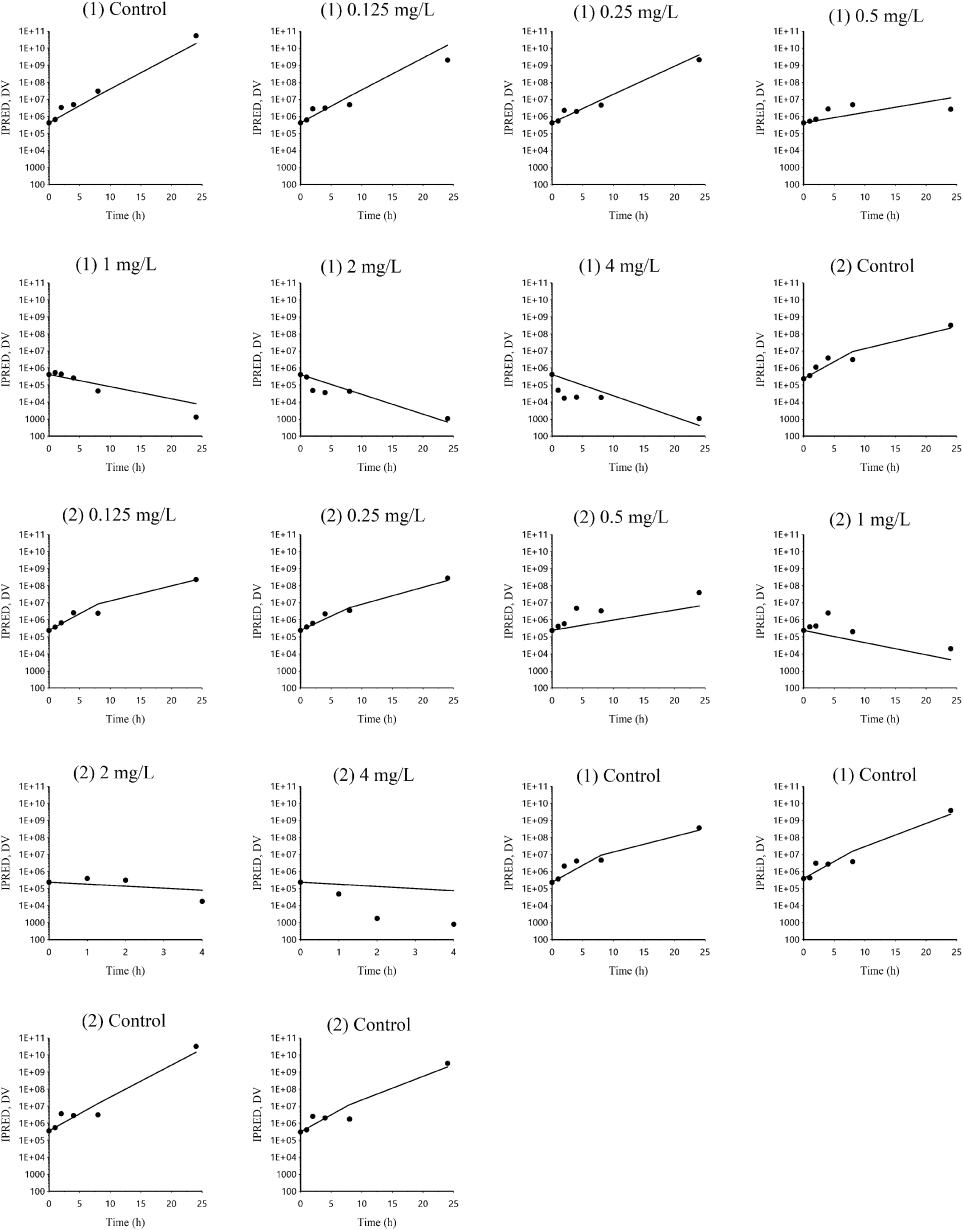
**Plot (latticed by individual) of dependent variable (DV) i.e. observed bacterial concentration (black spots) and IPRED i.e. individual model predicted bacterial concentration (black line) of S. delphini vs time (h).** DV: dependent variable i.e. observed bacterial concentration CFU/mL, IPRED: individual model predicted natural logarithm of bacterial concentration (CFU/mL). There were two *S. delphini* isolates at each TYL concentration in duplicate (the average of viable cell counts was used for each isolate), except for the control where the same two isolates were tested in triplicate. The two isolates were indicated by number 1 and 2 in brackets corresponding to the isolates ID of 16-12727-1 and 16-12403-3, respectively.

Plots of observed and individual predicted bacterial population vs. time (latticed by isolate and drug concentrations) are presented in Figure 3, and plots of observed vs. individual model predicted bacterial population are presented in Additional file 2. Estimated primary and secondary parameters for the time-kill model are presented in Table 1. The degradation rate of TYL (k_el_) and the bacterial natural death rate in the absence of TYL (k_death_) were fixed to 0 (i.e. no degradation of the drug during 24 h in test tubes) and 0.17 1/h (a classical default value corresponding to a half-life of 4.08 h [15]), respectively. The maximal bacterial killing rate (E_max_) of TYL was 0.75 1/h, yielding a 4.4 fold increase in fixed spontaneous death rate. To achieve half of the maximal killing effect (EC_50_), a TYL concentration of 0.55 mg/L was required. The MIC and MBC estimated by the model were 0.36 and 1.45 mg/L, respectively. Considering the inherent methodological limitation of MIC measurements (i.e. an MIC may vary two-fold up and down between repeated measurements), the estimated MIC of 0.36 mg/L predicted by the model was in good agreement with the MIC measured by in vitro broth microdilution (0.25 mg/L).

**Table 1:** Estimation of primary and secondary PD parameters obtained from a model of the time kill experiment of TYL against *S. delphini*

Parameter	Units	*S. delphini*	
		Mean	SE
Primary parameters			
K_el_	1/h	0 (fixed)	
K_growthmax_	1/h	0.63	0.017
K_death_	1/h	0.17 (fixed)-	
B_max_	CFU/mL	4.8 × 10^9^	4.2 × 10^8^
E_max_	1/h	0.75	0.022
Gamma	Scalar	2.73	0.275
EC_50_	mg/L	0.55	0.026
SD	Scalar	1.16	0.079
IIV B_max_ (CV%)		878	0.604
η shrinkage B_max_		0.02	
Secondary parameters			
MIC	mg/L	0.36	0.023
MBC	mg/L	1.45	0.049

K_el_ is the drug degradation rate constant fixed as 0 to express no degradation of TYL during 24 h in a test tube, K_growthmax_ is the maximum growth rate constant of bacterial strains, K_death_ is the natural death rate constant of a bacterial strain that is equally present in the S and P compartments and was considered at a fixed rate of 0.17 per hour, B_max_ is the total bacterial count in the system (S + P), E_max_ is the maximal effect of drug against bacterial species assessed by a maximal killing rate (efficacy), gamma (γ) is the curve slope, EC_50_ is the concentration of drug that produces half of the maximal effect, SD is standard deviation of the residual (exponential model for the control curves), IIV is inter-individual (i.e. curve) variability expressed as coefficient of variation (CV%), this variability corresponded to estimated post-hoc values of B_max_ from 1.86 × 10^8^ to 3.58 × 10^10^ for the 6 curves, Shrinkage refers to the quality of the estimated IIV, MIC and MBC are minimum inhibitory and bactericidal concentration of drug against bacterial species as estimated by the model. The precision of parameters (SE) was computed by the bootstrap tool in Phoenix^®^.

### The PK parameters and disposition of TYL in mink

**Figure 4:**
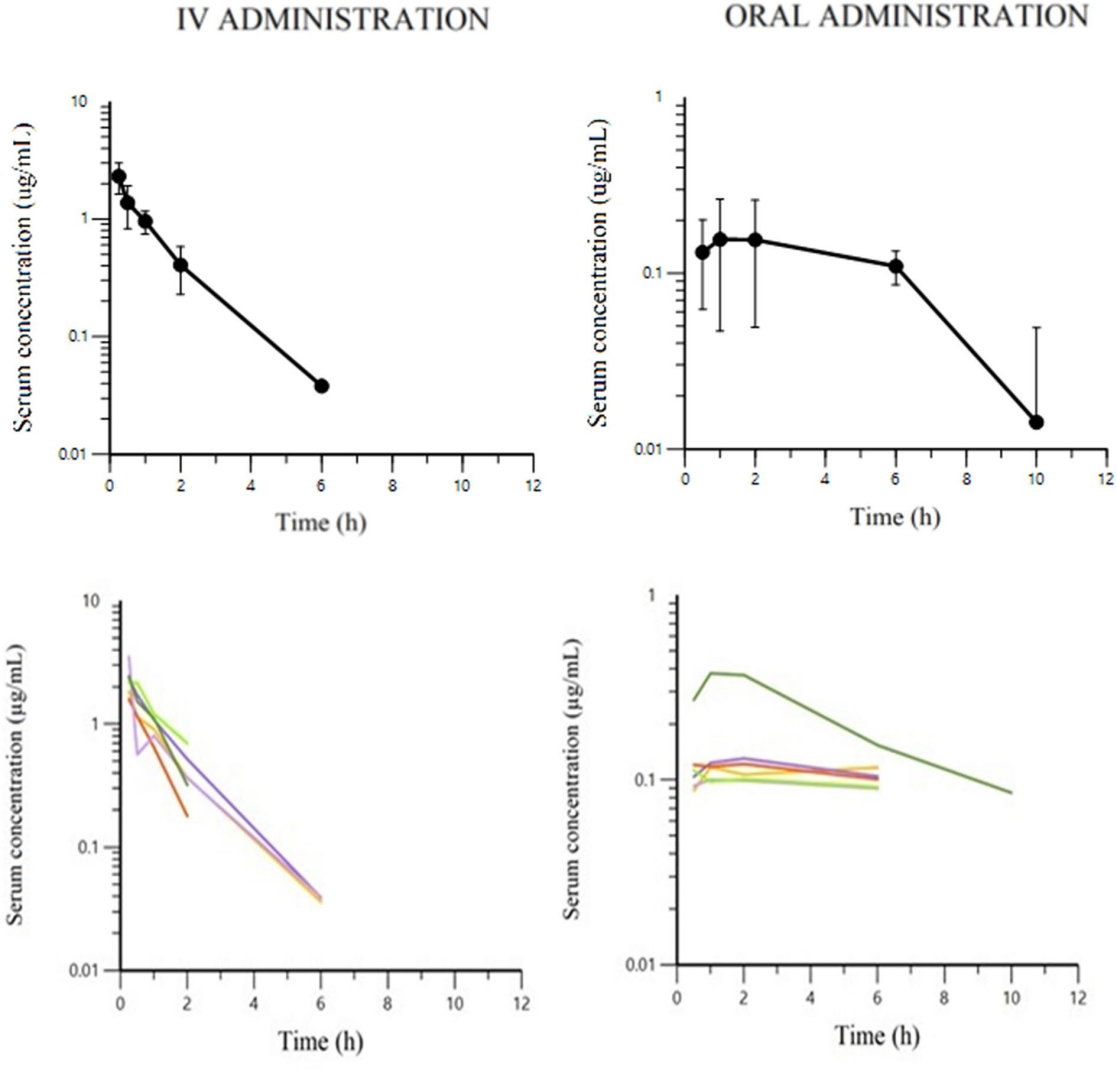
**Semi-logarithmic plots of mean ± SEM (top) and spaghetti plots (bottom) of serum TYL concentration after IV (left) and oral (right) administration (LLOQ = 30 ng/mL).**

**Figure 5:**
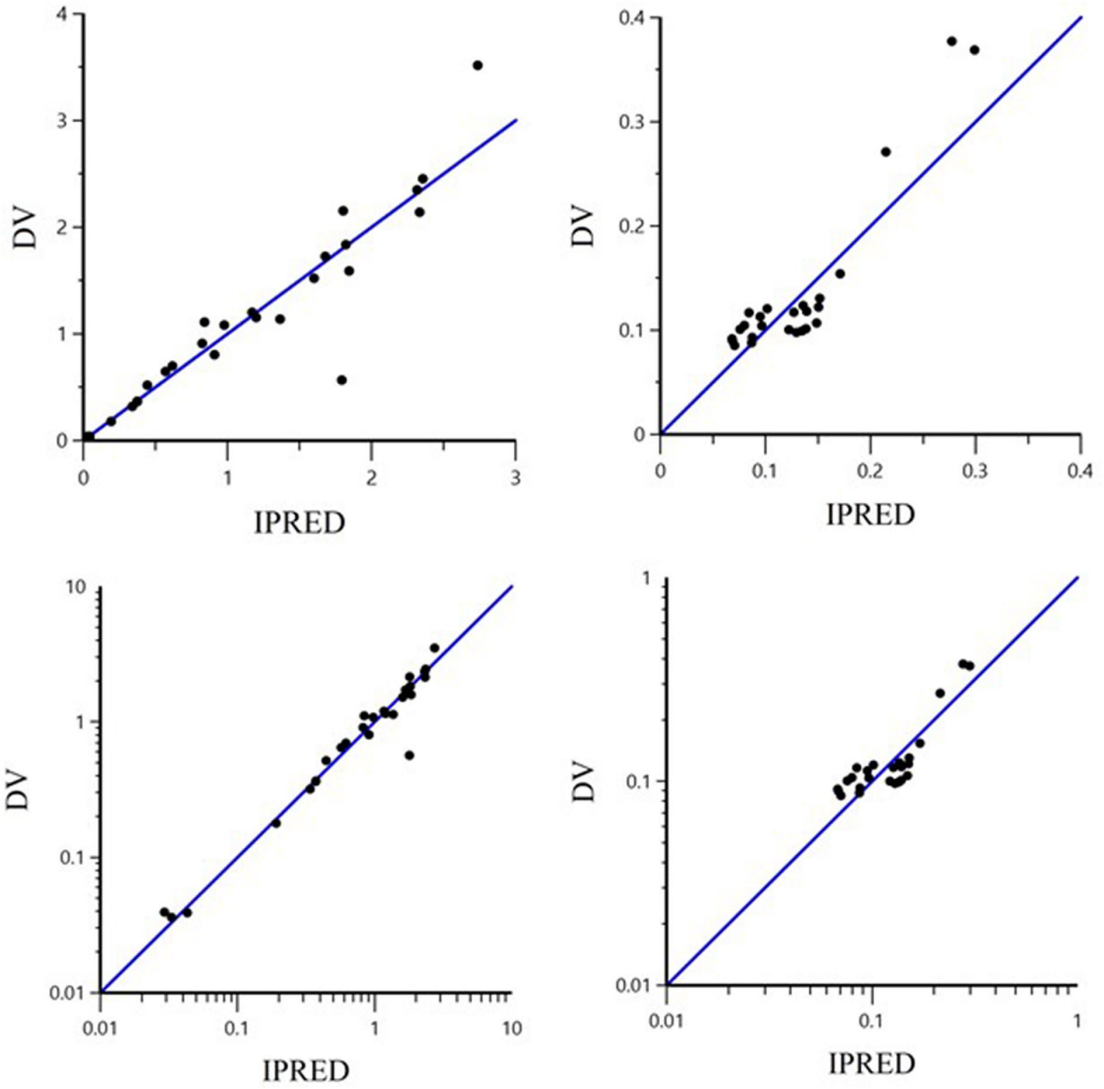
**Plot of the dependent variable (DV) i.e. observed TYL serum concentrations (µg/mL) versus individual predicted (IPRED) serum concentrations in arithmetic (top) and logarithmic (bottom) scale.** The IPRED plots were obtained by setting random effects to the “post hoc” or empirical Bayesian estimate of the random effects for the individual from which the dependent variable (DV) observation was made. Thus, the plots show observed vs fitted values of the model function. Ideally, points should fall close to the line of unity y = x.

**Figure 6:**
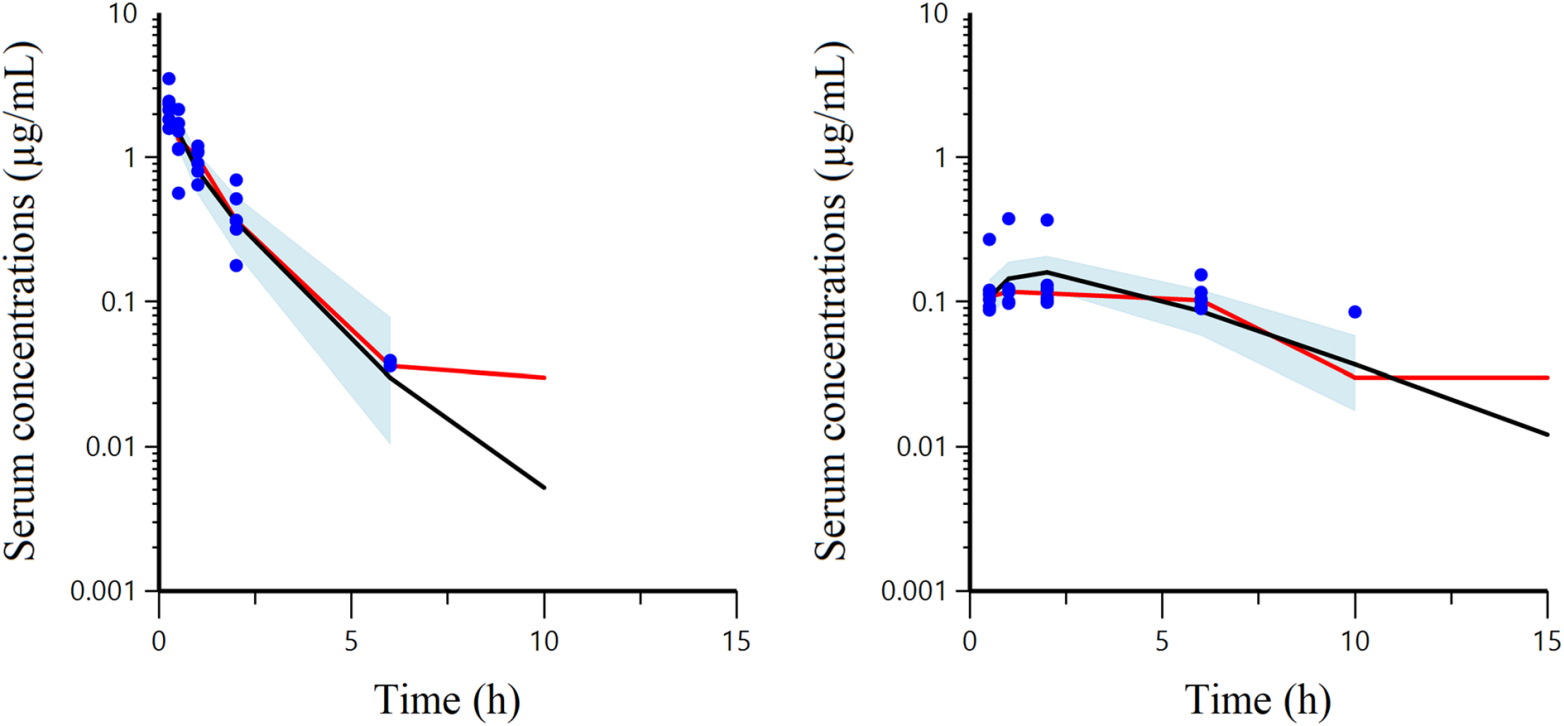
**Visual Predictive Check (VPC) plots of observed serum concentration (µg/mL) of TYL vs. time (h) and observed and predicted quantiles.** The VPC diagnostic plots illustrate the trends with the observed 50% quantiles (red line) and predictive check 50% quantile (black line) that were computed using Monte Carlo simulation over the observed serum concentrations (blue spots) following IV (left) and oral (right) administration. The blue shaded area corresponds to the 95% confidence interval of the 50% simulated quantile (black line).

The semi-logarithmic plots of TYL disposition in mink serum after IV and oral administration of 10 mg/kg body weight are presented in Figure 4. The dependent variable (DV) i.e. observed serum concentration (µg/mL) vs. individual prediction (IPRED) of concentration (µg/mL) plots in arithmetic and logarithmic scale after IV and oral administration are presented in Figure 5. Visual inspection of the plots shows that the observed and predicted points are close to the line of unity (x = y), except for some data points with high TYL concentrations after oral dosing. The visual predictive check (VPC) plots for different administration modalities are presented in Figure 6. Plots of observed and individual predicted serum concentration vs time after dosing (latticed by individual) are presented in Additional file 3. The NLME-estimated primary and secondary PK parameters following IV and oral administration are presented in Table 2. The bioavailability of TYL was 41% with the peak plasma concentration (C_max_) of 0.43 mg/L observed 1.75 h (T_max_) after oral administration of 10 mg/kg body weight. The CL and volumes of distribution (V_1_ and V_2_) were 3.41 L/h/kg, 2.24 and 1.33 L/kg, respectively, indicating a very high body clearance and extensive distribution of TYL into the tissues. The multiplicative component of the residual error was rather low as demonstrated by the CV of 15%. The inclusion of the sample site (i.e. covariate) into the model for the plasma clearance did not reduce the BIC, and it was concluded that the sampling site (vein vs nail) had no influence on the plasma clearance. Hence, the clearance was estimated ignoring a possible effect of sampling site.

**Table 2:** Population primary and secondary parameters of TYL estimated with a two-compartment model by fitting IV and oral PK data simultaneously

	Parameter	Units	IV and oral	
			TV	SE
Primary parameters	Ka	1/h	0.23	0.023
	V_1_	L/kg	2.24	1.076
	V_2_	L/kg	1.33	0.523
	CL	L/h/kg	3.41	0.756
	Q	L/h/kg	1.97	1.453
	F		0.41	0.150
	MultiSD	Scalar	0.15	0.114
	SD IV	µg/mL	0.020	0.007
	SD Oral	µg/mL	0.006	0.006
	BSV V_1_ (CV%)		21.21	0.079
	BSV CL (CV%)		10.00	0.161
	η shrinkage V_1_		0.12	
	η shrinkage CL		0.20	
Secondary parameters	AUC_IV_	µg h/mL	3.05	0.565
	AUC_Oral_	µg h/mL	1.18	0.195
	Ka _HL_	h	3.00	0.319
	T_max_	h	1.75	0.138
	C_max_	µg/mL	0.43	0.072

Ka is the absorption rate constant, V_1_ and V_2_ are volumes of distribution in central and peripheral compartments, respectively, CL is body clearance, Q is inter-compartment clearance, MultiSD is multiplicative error term and it should be interpreted as a coefficient of variation, (here of 15%), SD is the additive component of the error term, BSV is between subject variability expressed as CV, Shrinkage refers to the quality of the estimated BSV. For the present study, an η-shrinkage lower than 0.4 has been considered as acceptable. AUC_IV_ and AUC_Oral_ are area under the concentration–time curve after IV and oral dosing, respectively and Ka_-HL_ is half-life of absorption. TV is the population typical value. The precision of parameters (SE) was computed by the bootstrap tool in Phoenix^®^.

### Prediction of the PKPD index and PKPD target

**Figure 7:**
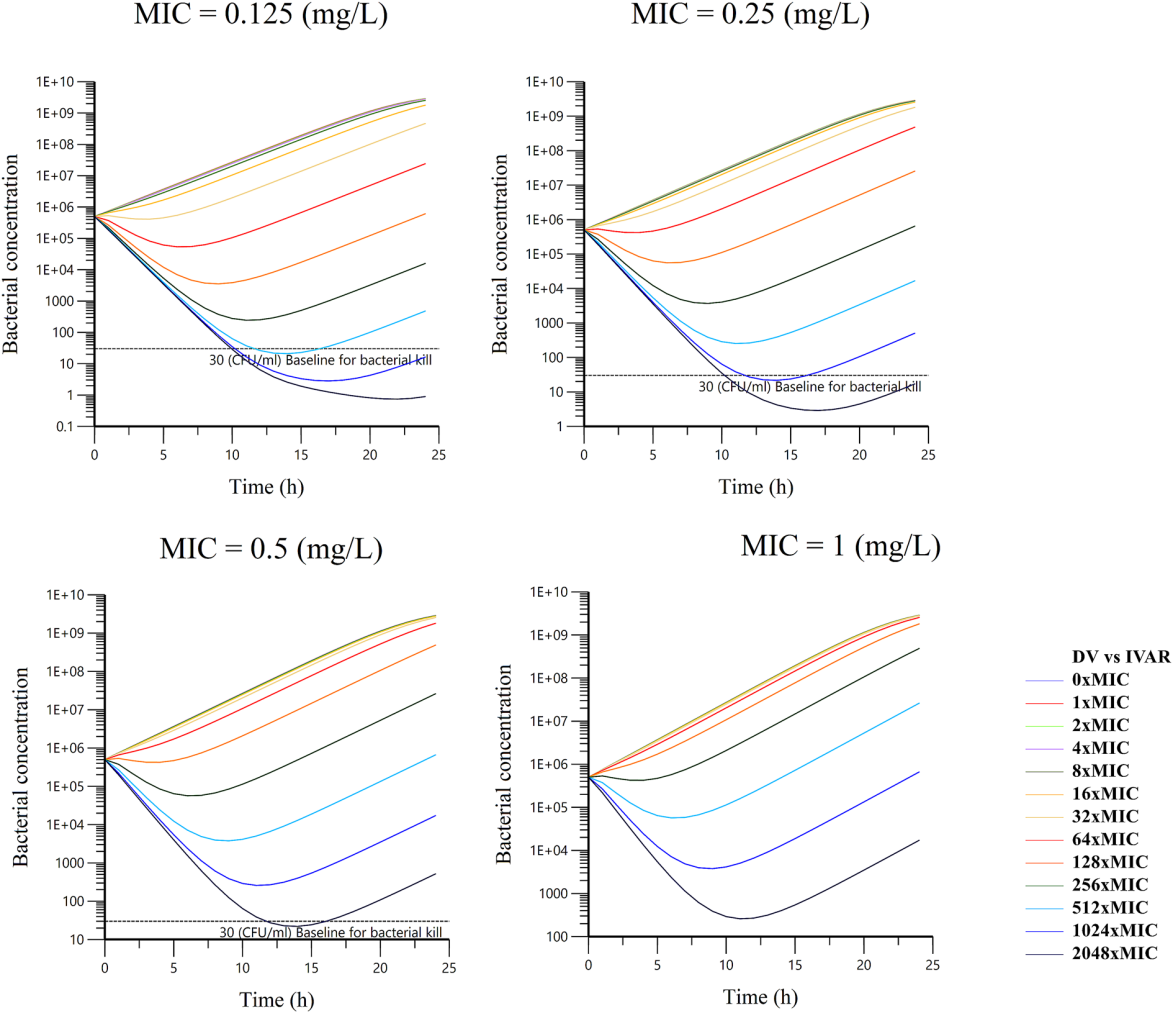
**Escalating dose plots of 13 daily oral TYL doses against S. delphini displaying four different TYL MICs.** The x-axis represents time after dosing (h) and the y-axis represents total bacterial concentration (CFU/mL). The baseline for bacterial kill (30 CFU/mL) was arbitrarily defined as the baseline for bacterial concentration that can be eliminated by the immune system.

**Figure 8:**
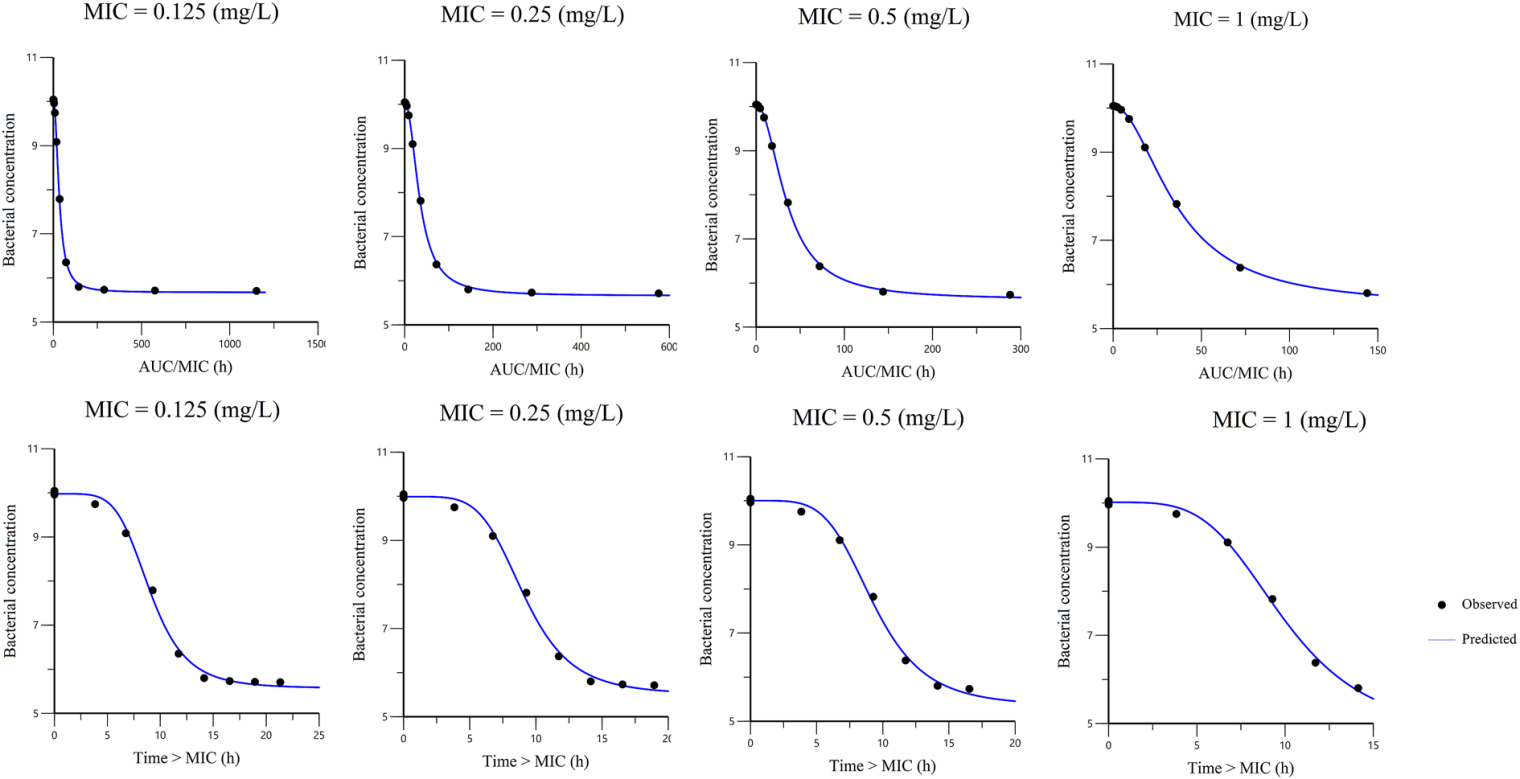
**The observed vs. predicted values of the I**_**max**_** Sigmoidal model for fAUC/MIC (top) and fT > MIC (bottom) of TYL against S. delphini displaying four distinct TYL MICs.** The y-axis represents the Log_10_ bacterial concentration (CFU/mL), and the x-axis represents different values for the PKPD index (*f*AUC/MIC and *f*T > MIC).

Escalating dose plots of TYL against *S. delphini* displaying four distinct MICs are presented in Figure 7. The simulated initial inoculum was fixed to 5 × 10^5^ CFU/mL, and the final concentration of 30 CFU/mL was arbitrarily selected as the endpoint for a full bactericidal effect. Below this value, there was no possible regrowth and curves were truncated for computation of effects. The visual inspection of plots revealed that extremely high doses of TYL would be required to reach the bacterial baseline of 30 CFU/mL. The initial (time = 0) minimum required concentrations of TYL for *S. delphini* to reach the bacterial concentration baseline after 24 h with MICs of 0.125, 0.25 and 0.5 mg/L were 512, 1024 and 2048 × MIC, respectively, due to the rapid in vivo disappearance of TYL (see Discussion). The in silico simulations of killing upon TYL exposure allowed a comparison of the two possible PKPD indices, namely *f*AUC_po,0-24 h_/MIC and *f*T(%) > MIC. The curves are presented in Figure 8. Based on visual inspection of curves, there was a slightly better fit of data for *f*AUC_po,0-24 h_/MIC compared to *f*T(%) > MIC, however the difference is not very noticeable. Table 3 presents the parameters of the I_max_ model and goodness of fit values for an initial inoculum of 5 × 10^5^ CFU/mL. The mean index_50_ for four MIC levels was 48.9 h for *f*AUC_po,0−24 h_/MIC, indicating that the required in vivo concentration of TYL to achieve half of the maximal bacteriostatic effect, should be at least 2.04 fold the MIC (a scalar factor corresponding to the index_50_, divided by 24 h). The mean index_50_ for *f*T(h) > MIC was 12.1 h, meaning the in vivo concentration of TYL should be above the MIC for at least 50% of the time during 24 h to achieve half of the maximal bacteriostatic effect. Based on the 28.4 points reduction in AIC for *f*AUC_po,0−24 h_/MIC compared to *f*T(%) > MIC (Table 4), the former PKPD index was selected a priori for predicting the effect of TYL against *S. delphini*. The target values for *f*AUC_po,0−24 h_/MIC and *f*T(%) > MIC are presented in Table 4. From simulations using four different MICs, the mean values for *f*AUC_po,0−24 h_/MIC with bacteriostatic and bactericidal effect of TYL against *S. delphini* were 48.9 and 98.7 h, respectively. To express the PKPD index as a scalar value without dimension, these values were divided by 24 h [16], yielding 2.04 and 4.11 for bacteriostatic and bactericidal effect, respectively. This indicates that, in vivo average TYL plasma concentrations of two- and fourfold the MIC are required over a 24 h treatment interval to exert bacteriostatic and bactericidal effects, respectively.

**Table 3:** Mean and SE of estimated I_max_ sigmoidal model parameters for *f*AUC/MIC and *f*T > MIC of TYL against MICs in *S. delphini*

Parameters	*f*AUC_po,0−24 h_/MIC (h)	*f*T>MIC (h)
I_max_		
Mean	4.15	5.05
SE	0.09	0.15
Index_50_		
Mean	48.95	12.06
SE	1.66	0.15
E_0_		
Mean	10.47	10.43
SE	0.02	0.01
Gamma		
Mean	1.75	3.50
SE	0.08	0.14
AIC	−57.8	−29.4
R^2^	0.9997	0.997

I_max_ (Log_10_ transformation of bacterial concentration (CFU/mL)) is magnitude of the maximal effect of TYL, the index_50_ is magnitude of the PKPD index (*f*AUC_po,0−24 h_/MIC (h) or *f*T > MIC (h)) that achieves 50% of the maximal effect, E_0_ (Log_10_ transformation of bacterial concentration (CFU/mL)) is maximum effect in the absence of antimicrobial (obtained from control group), and gamma (γ) is the sigmoidicity factor, reflecting the steepness of the relationship. AIC is Akaike’s information criterion, with lower AIC indicating better fit of data to the model. R^2^ is the coefficient of determination, indicating the amount of variation explained by the model.

**Table 4:** Calculated PK/PD targets for a bacteriostatic and bactericidal effect, respectively, of TYL against *S. delphini*

	*f*AUC_po,0–24 h_/MIC (h)		(*f*AUC_po,0–24 h_/MIC)/24 Scalar		*f*T > MIC (h)		*f*T > MIC (%)
	B_static_	B_cidal_	B_static_	B_cidal_	B_static_	B_cidal_	B_cidal_
Average	*48.91* (± 0.3)	*98.72* (± 0.9)	*2.04* (± 0.01)	*4.11* (± 0.04)	*10.30* (± 0.04)	*13.20* (± 0.06)	*55* (± 0.26)

The value for *f*AUC _po,0–24_/MIC (h) presents the required area under the concentration–time curve of TYL over MIC, and *f*T > MIC (%) is the required duration of serum concentration of TYL above MIC. The PKPD targets were obtained (mean ± SE from an I_max_ sigmoidal model for predicted bacteriostatic and bactericidal effects corresponding to no change and one Log_10_ reduction, respectively, of initial bacterial concentration after 24 h. Scalar *f*AUC/MIC was obtained by dividing the AUC/MIC cutoff by 24 h.

### Prediction of TYL dosage regimen

Considering the median of body clearance of 3.41 L/kg/h, the oral bioavailability of 41%, a mean free drug proportion of 69% (Table 2), the TECOFF of 2 mg/L [14] and the 4.11 target value of *f*AUC/MIC for TYL against *S. delphini* for a bactericidal effect (Table 4), the computed daily oral dosage was 2378 mg/kg body weight using Eq. (9). For a bacteriostatic effect of TYL, considering the PKPD target of 2.04, the calculated daily dosage was 1180 mg/kg. By using Monte Carlo Simulation (MCS), and the PK population model, PKPD cutoffs for the current empirical dosage of 10 mg/kg TYL once a day i.e. the maximal achievable MIC in a given percentage of the mink population [17] were 0.004 and 0.008 µg/mL with probability of target attainment (PTAs) of 90% and 50%, respectively.

## Discussion

Very few antimicrobial agents are registered for mink, hence most antimicrobial use in this species has to follow the cascade principle. This principle allows prescribing antimicrobials for use other than indicated in the product information. Such off-label use of antimicrobials is typically based on limited or no evidence for the target animal species and may therefore be accompanied by negative consequences including treatment failure and selection of antimicrobial resistance [18]. According to a recent recommendation by the European Medicines Agency (EMA), strategies to fight antimicrobial resistance should encourage the development of existing antimicrobials for use in minor species [19]. Our assessment of the validity of the current empiric TYL dosage regimen used in mink (10 mg/kg) is in line with that and with a recent recommendation to use a PKPD approach for optimizing dosage regimens of old antimicrobial agents with limited available clinical data [20].

Overall, the disposition of TYL in mink upon oral administration of 10 mg/kg via feed was characterized by a relatively poor oral bioavailability, an extensive distribution and, overall, by a very high body clearance leading to plasma concentrations, which were always lower (C_max_ of 0.43 mg/L) than the *S. delphini* TECOFF of 2 mg/L [14]. Gastrointestinal absorption of TYL is generally rapid in monogastric animals with plasma concentrations peaking 1–3 h after oral dosing [21]. In line with this, we observed a peak plasma concentration already after 1.75 h. The poor bioavailability of TYL in mink (41%) is comparable to the 23 and 35% bioavailability of this drug reported in pigs [22] and chicken [23, 24], respectively. These rather low percentages might be due to the instability of TYL in acidic milieu like that of the stomach [25]. The steady-state volume of distribution (i.e. V1 + V2) was 3.5 L/kg i.e. comparable to the findings in duck (2.29 L/kg) [26] and chicken (2.67 L/kg) [27]. This extensive tissue distribution is a common trait of macrolides, which as weak bases become entrapped in cells that are more acidic than plasma, e.g. in the cells of lung, liver, kidney and heart [21, 28]. The most noticeable characteristic of TYL disposition in mink was the very high body clearance (3.41 L/h/kg). The AUC is controlled by body clearance and bioavailability following extravascular administration of a drug [29]. The rapid clearance and poor bioavailability of TYL in mink resulted in an AUC/dose ratio (0.331 and 0.091 µg h mL^−1^/mg.kg^−1^ after IV and oral administration, respectively) about three times lower than reported in chicken (0.25 µg h mL^−1^/mg kg^−1^ after oral administration) [23] and duck (0.3 µg h mL^−1^/mg.kg^−1^ after oral administration) [26], and 14 times lower than in dog (4.74 µg h mL^−1^/mg kg^−1^ after an IV administration) [30]. This means that a much higher dose of TYL would be required in mink compared to these other animals in order to reach the same AUC levels.

In the first in vivo study on drug disposition after IV administration, we withdrew blood from both the cephalic vein and nails of mink in order to compare PK results and assess whether nail blood may replace venous blood, which is typically used to analyze drug disposition. There was no statistical difference in the computed clearance between venous and nail blood samples obtained in parallel when using a model with and without a covariate for the sampling site. Therefore, to avoid the additional step of anesthesia, we decided to take only nail blood in the subsequent in vivo study on TYL bioavailability.

Beyond PK properties, the second component of a dosage regimen is the drug PD properties. To deal with the intrinsic shortage of the MIC value, namely its failure to elucidate dynamic rather than static effects of an antimicrobial during exposure [11, 31, 32], an in vitro semi-mechanistic model for analysis of time-kill experiments was used in this study. In this PD model, a random component was added in the model for B_max_ for each tested dose level, because B_max_ structurally reflects differences between the strains (such as K_growth_), and in addition because B_max_ is directly observable for each curve and its value has been estimated with low shrinkage. Also, the inclusion of the random component on B_max_ improved the precision of the estimations for pharmacodynamic parameters. This model allowed us to obtain the three genuine PD parameters, namely efficacy, potency and sensitivity of TYL against *S. delphini* (Table 1). In addition, PKPD modeling based on the in vitro time-kill experiment and in vivo TYL disposition in mink, offered an alternative approach to select and estimate the PKPD index instead of the traditionally used in vivo rodent models with concurrent ethical and financial issues. In general, macrolides are considered time-dependent antimicrobials, but for some macrolides producing a prolonged persistent effect (e.g. azithromycin) the ƒAUC/MIC is an appropriate predictor of effect and can be used as PKPD index [6, 13, 33, 34]. The selection of *f*AUC/MIC as PKPD index in this study was based on AIC reduction (Table 3) and was in agreement with previous studies on TYL in pig [35] and duck [26]. In our simulations for predicting a PKPD target, we selected 30 CFU/mL as a lower limit where no bacterial re-growth would be possible to capture all the curves of bacterial evolution, although for a non-immunocompromised subject, the natural process of bacterial eradication by the immune system can be efficient up to 10^5^ CFU/mL [36]. We selected the one-log_10_ reduction of the initial bacterial concentration as a relevant endpoint of efficacy for the in vivo situation to estimate the PKPD target. This is a univocal efficacy endpoint that is targeted in human medicine [37]. The estimated *f*AUC_po,0−24 h_/MIC for bacteriostatic and bactericidal effects was 48.9 and 98.7 h, respectively. It has been suggested to divide ƒAUC/MIC by 24 h to generate a scaling factor without a time dimension. This scaling factor simplifies the understanding of ƒAUC/MIC and has clinical application [16]. In this study, the scaling factors for bacteriostatic and bactericidal effects of TYL were 2.04 and 4.11, respectively. These scaling factors mean that the goal for a rational dosage is to maintain a plasma concentration equal to 2 or fourfold the MIC to comply with PKPD results, and these figures can be directly incorporated into the dosage Eq. (9) for daily dose determination [16].

Using this approach, we computed extremely high and unachievable oral dosages of 2378 and 1180 mg/kg for bactericidal and bacteriostatic effects, respectively, to target the *S. delphini* wildtype population with TYL MICs equal to or lower than the TECOFF of 2 mg/L. As explained above, this high dosage is a result of the very high clearance and low bioavailability of TYL in mink combined with a rather high TYL TECOFF of 2 mg/L for *S. delphini*. By considering the PKPD cutoffs of 0.008 and 0.004 µg/mL for the currently used dosage of 10 mg/kg with 50% and 90% probability of target attainment in mink populations, respectively, and by comparing these cutoff values with the TECOFF of 2 mg/L for *S. delphini*, it can be deduced that at least 250 and 500 times the currently used dosage of 10 mg/kg TYL would be required for treatment of *S. delphini* infections. The very high calculated dose in mink is consistent with the fact that in chickens an oral dose of 100 mg/kg/day does not allow plasma concentrations to exceed 0.4 µg/mL despite the oral clearance in chickens being 3 times lower than in mink [38].

It should be noted that the calculated dose may be higher than actually needed due to a possible bias when measuring MICs. In that regard, it has recently been shown that the MICs of tulathromycin for *Mannheimia haemolytica* and *Pasteurella multocida* were 50 times lower in calf serum than in MHB [39]. Accordingly, it cannot be excluded that the TECOFF measured in MHB was overestimated compared to what it would have been using serum. Another point is the TYL anti-inflammatory properties, which can modulate COX-2 and iNOS gene expression and the production of cytokines by immune cells [40]. Although this boosting effect on the immune system and a potential effect of serum may partially contribute to a clinical effect of TYL in minks, these factors are not likely to reduce the TYL dose to a sufficiently low level. The same is true when taking into consideration other potential target pathogens, e.g. *Streptococcus canis*, which is a less common cause of infections (e.g. of the skin) in mink [2]. Although this opportunistic pathogen has a lower TECOFF (0.25 mg/L) than *S. delphini* [14], very high dosages of 297 and 148 mg/kg would be needed for bactericidal and bacteriostatic effects against *S. canis*. It should be noted that these calculations are associated with some uncertainty, as they are based on the same PKPD target as determined for *S. delphini*. One potential limitation in our in vivo experiments is the use of an analgesic and anesthesia, which was necessary for practical and ethical reasons. The use of such drugs may influence the disposition of TYL due to alterations of biological functions like cardiac output and blood flow to internal organs [41]. Although this evidence relies on studies in humans, we cannot exclude that meloxicam and sevoflurane affected TYL disposition in mink and consequently our results. However, these possible effects are not likely to question our conclusions that TYL cannot be used to treat a systemic infection in mink when the MIC of a wild-type population is as high as 2 mg/L.

Using a PKPD approach, TYL can be considered either as a concentration or a time-dependent antimicrobial agent for *S. delphini*. Considering TYL as concentration-dependent, a bacteriostatic and bactericidal effect against *S. delphini* can be obtained with *f*AUC_po,0−24 h_/MIC of 48.9 h and 98.7 h, respectively. The daily dosage of TYL against this pathogen was predicted to be 2378 and 1180 mg/kg for bactericidal and bacteriostatic effects, respectively. Although the wildtypes of other mink pathogens such as *S. canis* have lower MICs, required dosages are notably higher than the currently used off-label dosage of 10 mg/kg, and these dosages are simply impossible to reach in practice due to the risk of toxicity. The PKPD cutoff for the currently used dosage of 10 mg/kg once a day was 0.004 mg/L with PTA 90%. Therefore, the current TYL dose for treatment of extra-intestinal mink infections is not supported by PKPD data and should be avoided to minimize the risk of treatment failure and selection of antimicrobial resistance.

## Supplementary Information

**Additional file 1. Quantification of TYL in samples.**

**Additional file 2. Plots for PD modeling of TYL against** ***S. delphini.***

**Additional file 3. PK plots obtained from NLME.**

## Data Availability

The datasets and model files generated during the current study were created in the Mendeley Data platform, http://dx.doi.org/10.17632/wxc782dhkd.1

## Abbreviations

AIC: Akaike’s Information Criteria
Alpha-_HL_: Distribution half-life
AUC: Area under the time–concentration curve
Beta-_HL_: Elimination half-life
BQL: Below Quantification Limit
B_max_: Maximum achievable bacterial concentration in the system
BSV: Between subject variability
CL: Body clearance
C_max_: Maximal serum concentration
DV: Dependent Variable
(T)ECOFF: (Tentative) Epidemiological cut off
E_0_: Effect at the baseline
EC_50_: The concentration of drug that produces half the maximum effects
ETA (η): Random effect describing the deviation of the individual empirical Bayes estimate of the parameter from the typical population parameter estimate
*f*: Free fraction of drug
F: Bioavailability of drug
Gamma (γ): Hill coefficient, sigmoidicity factor
IC_50_: Concentration producing half the maximum inhibitory effect
IPRED: Individual predicted value based on individual’s ETAs
I_max_: Maximum inhibitory effect
Ka: Absorption rate constant
Ka_-HL_: Absorption half-life
K_death_: Bacterial natural death rate constant
K_el_: Degradation constant of drug in the test tube condition
K_growth_: Bacterial growth rate constant
K_growthmax_: Maximum achievable bacterial growth constant
LLOQ: lower limit of quantification
MBC: Minimum bactericidal concentration
MHA: Muller Hinton Agar
MHB: Muller Hinton Broth
MIC: Minimum inhibitory concentration
OMEGA (ω^2^): Matrix of between subject variance
PD: Pharmacodynamics
PK: Pharmacokinetic
Q: Inter-compartmental clearance
SF: scaling factor
SIGMA (σ^2^): Residual variance
TAD: Time after dosing
T_max_: Time at maximum serum concentration
TYL: Tylosin
V_1_: Volume of distribution in central compartment
V_2_: Volume of distribution in peripheral compartment
VPC: Visual predictive check
