# Supplementary material for: Evaluating a tylosin dosage regimen for treatment of Staphylococcus delphini infection in mink (Neovison vison): a pharmacokinetic-pharmacodynamic approach
Source: Vet Res. 2021 Feb 27;52:34. doi: 10.1186/s13567-021-00906-0 (PMC7913401; doi:10.1186/s13567-021-00906-0)
Supplement: Supplementary file 1 — Additional file 1. Quantification of TYL in samples. [file 13567_2021_906_MOESM1_ESM.docx]

# **Additional file 1 Quantification of TYL in samples**

**Materials and methods**

Acetonitrile, methanol, formic acid and 25% ammonium hydroxide, all of LC-MS grade and ethyl acetate were obtained from Sigma Aldrich (Schneldorf, Germany). Isolute bulk C-18 sorbent was obtained from Biotage (Uppsala, Sweden), the sorbent was washed with acetonitrile and ethyl acetate and dried prior to use. Water was purified on a Milli Q system (Millipore Corporation, MA US). Tylosin was purchased from Sigma Aldrich and stock solutions of 1 mg/ml were prepared in methanol.

**Sample preparation**

Fifty µL of mink plasma was transferred to an Eppendorf vial and added 150 µL of ice cold methanol. 50 mg of isolute C-18 sorbent was added to the tube which was shaken for 5 min, cooled to 4 °C and centrifuged at 10 000 × *g* for 10 min at 4 °C. 50 µL of the supernatant was transferred to an HPLC vial and mixed with 150 µL of 50% methanol prior to analysis by LC-MS.

**Liquid Chromatography Quadrupole Time of Flight Mass Spectrometry (LC-QtoF-MS)**

Liquid Chromatography was performed on a Dionex Ultimate 3000 RS (Thermo Scientific, CA, USA) with a Poroshell SB C-18 (100x2.1 mm, 2.7 µm particle size) column held at 25 °C (Agilent technologies, Waldbronn, Germany). The solvent system consisted of A: 2.5 mM ammonium hydroxide + 0.1 % formic acid in water and B: acetonitrile. Solvent programming were: 2% B from 0 to 1 min followed by a linear gradient to 50% B to 8 min and a linear gradient to 98% B to 10 min, isocratic 98% B from 10 to 12 min followed by reversal to initial conditions to 12.1 min and re-equilibration of the column to 15 min. The flow rate was 0.3 ml/min from 0 to 1 min followed by a linear gradient to 0.4 mL/min to 10 min, which was held to 12 min followed by reversal to initial conditions.

The LC system was connected to a Bruker Daltonics (Bremen, Germany), maXis qTOF mass spectrometer equipped with an electrospray ion source operated in positive ion mode (Bruker Daltonics). The ion source settings were: nebulizer pressure 2 bars, drying gas flow 10 l/min, dry gas temperature 200 °C, capillary voltage 4500 V. The scan range was from 80 to 700 m/z with an acquisition rate of 2 Hz. Sodium formate dissolved in 50% 2-propanol was introduced in the ion source in a 0.2-0.4 min time segment and used for internal calibration of the data files. Hexakisperflouroetoxyphosphazene was used as lock mass calibrant.

Matrix matched standard samples were prepared in blank plasma at 4 different levels in the concentration range of 4-500 ng/mL. Standards and blanks were analyzed in the beginning of a sequence and after each set of 20 samples.

**Data analyses**

Data files were processed using QuantAnalysis (Bruker Daltonics). Extracted ion chromatograms of m/z 916.5264 ± 0.005) Data were constructed and integrated. Plasma concentrations were calculated based on linear calibrations curves using 1/x weighing.
