# Supplementary material for: Evaluating a tylosin dosage regimen for treatment of Staphylococcus delphini infection in mink (Neovison vison): a pharmacokinetic-pharmacodynamic approach
Source: Vet Res. 2021 Feb 27;52:34. doi: 10.1186/s13567-021-00906-0 (PMC7913401; doi:10.1186/s13567-021-00906-0)
Supplement: Supplementary file 2 — Additional file 2. Plots for PD modeling of TYL against S. delphini. [file 13567_2021_906_MOESM2_ESM.docx]

**Additional file 2 Plots for PD modeling of TYL against *S. delphini***

Plots of dependent variable (DV) i.e. observed bacterial population vs. model-predicted individual bacterial population (IPRED) of S. delphini.


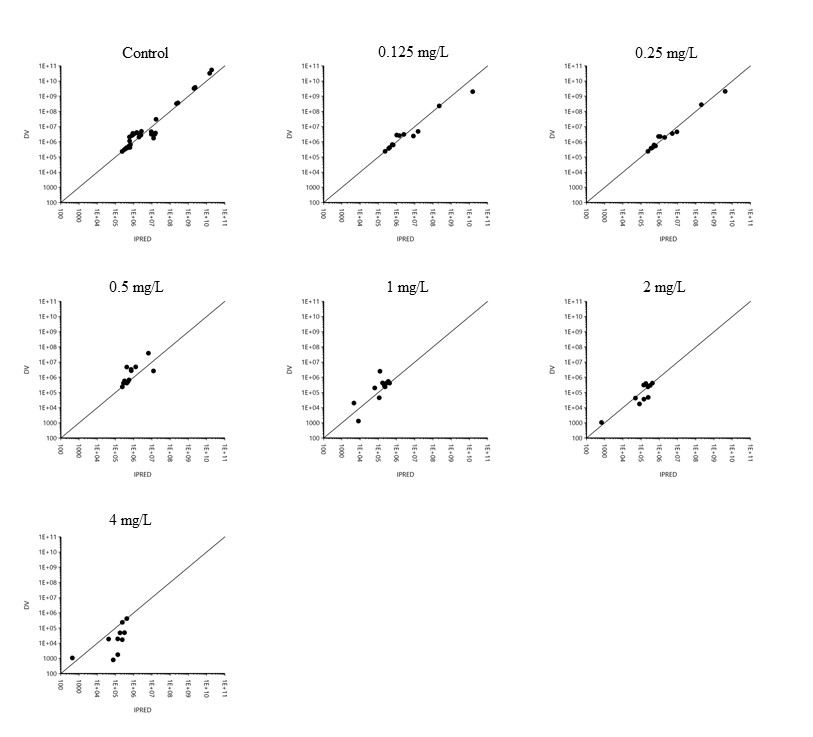


DV: dependent variable i.e. bacterial concentration (CFU/mL), IPRED: individual prediction of bacterial concentration (CFU/mL). There were two isolates for *S. delphini* at each TYL concentration, except for control that same two isolates were tested in triplicate.
