# Supplementary material for: Evaluating a tylosin dosage regimen for treatment of Staphylococcus delphini infection in mink (Neovison vison): a pharmacokinetic-pharmacodynamic approach
Source: Vet Res. 2021 Feb 27;52:34. doi: 10.1186/s13567-021-00906-0 (PMC7913401; doi:10.1186/s13567-021-00906-0)
Supplement: Supplementary file 3 — Additional file 3. PK plots obtained from NLME. [file 13567_2021_906_MOESM3_ESM.docx]

**
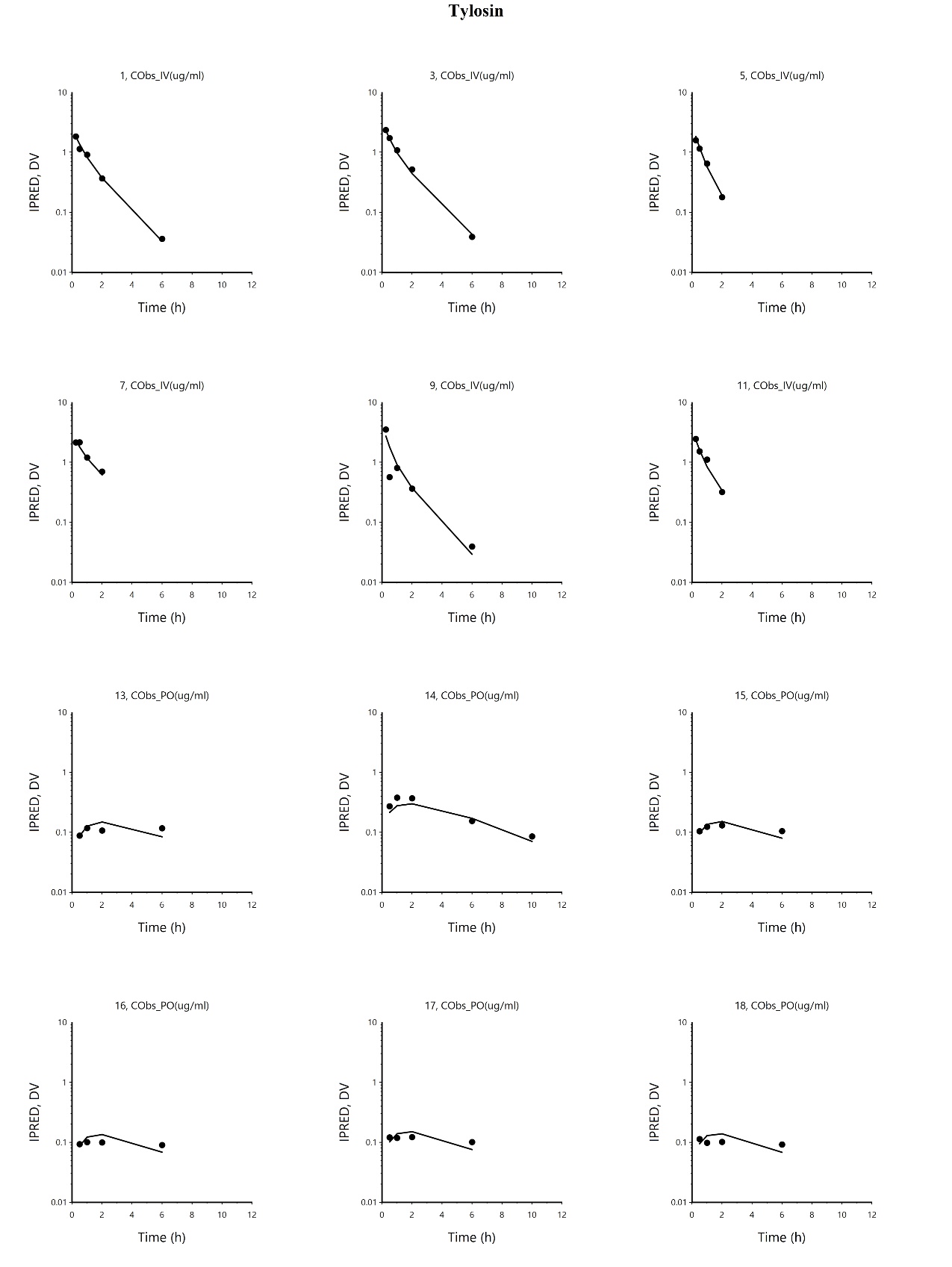
Additional file 3 PK plots obtained from NLME**

Plots (latticed by individual) of dependent variable (DV) i.e. observed TYL serum concentration (black spots), and individual predicted (IPRED) concentration (black line), vs time (h) after dosing (TAD), obtained from NLME.

***IPRED: Individual prediction of serum concentration, DV: dependent variable, TAD: time after dosing, IV: intravenous, PO: oral administration.***
